# Structural Basis of Chemokine Sequestration by a Tick Chemokine Binding Protein: The Crystal Structure of the Complex between Evasin-1 and CCL3

**DOI:** 10.1371/journal.pone.0008514

**Published:** 2009-12-30

**Authors:** João M. Dias, Christophe Losberger, Maud Déruaz, Christine A. Power, Amanda E. I. Proudfoot, Jeffrey P. Shaw

**Affiliations:** Merck Serono Geneva Research Center, Merck Serono S.A., Geneva, Switzerland; Griffith University, Australia

## Abstract

**Background:**

Chemokines are a subset of cytokines responsible for controlling the cellular migration of inflammatory cells through interaction with seven transmembrane G protein-coupled receptors. The blocking of a chemokine-receptor interaction results in a reduced inflammatory response, and represents a possible anti-inflammatory strategy, a strategy that is already employed by some virus and parasites. Anti-chemokine activity has been described in the extracts of tick salivary glands, and we have recently described the cloning and characterization of such chemokine binding proteins from the salivary glands, which we have named Evasins.

**Methodology/Principal Findings:**

We have solved the structure of Evasin-1, a very small and highly selective chemokine-binding protein, by x-ray crystallography and report that the structure is novel, with no obvious similarity to the previously described structures of viral chemokine binding proteins. Moreover it does not possess a known fold. We have also solved the structure of the complex of Evasin-1 and its high affinity ligand, CCL3. The complex is a 1∶1 heterodimer in which the N-terminal region of CCL3 forms numerous contacts with Evasin-1, including prominent π-π interactions between residues Trp89 and Phe14 of the binding protein and Phe29 and Phe13 of the chemokine.

**Conclusions/Significance:**

However, these interactions do not appear to be crucial for the selectivity of the binding protein, since these residues are found in CCL5, which is not a ligand for Evasin-1. The selectivity of the interaction would appear to lie in the N-terminal residues of the chemokine, which form the “address” whereas the hydrophobic interactions in the rest of the complex would serve primarily to stabilize the complex. A thorough understanding of the binding mode of this small protein, and its other family members, could be very informative in the design of potent neutralizing molecules of pro-inflammatory mediators of the immune system, such as chemokines.

## Introduction

Chemokines (chemotactic cytokines) are a subset of cytokines primarily responsible for controlling the cellular migration of various inflammatory cells. They compose a large family (approximately 40 known members in the human system)[1] of small proteins which share a relatively low level of sequence identity, but which display a remarkable structural homology, since they all contain the same monomeric fold. Chemokines control the migration of leukocytes through interaction with members of the G protein-coupled receptor family which possess seven transmembrane helices. The pairing of the chemokines to their receptors has been carried out, mainly by receptor binding assays, and has identified receptors that are specific (bind to a single ligand) or shared (bind more than one ligand). The association of certain receptors and ligands with disease has come from many studies of their expression in biopsy samples and body fluids, animal models and genetically engineered mice.

Dysregulation of the chemokine system can result in excessive cellular recruitment, with dramatic implications in inflammatory and autoimmune diseases[2]. Blocking the receptor-chemokine interaction should have therapeutic value, since prevention of this directional migration represents an effective anti-inflammatory strategy. Numerous reports in animal models have provided evidence to support this hypothesis using tools such as genetically engineered mice, neutralizing antibodies, and receptor antagonists[3]. However the most compelling data is in fact provided by nature - efficient strategies are employed by viruses and certain parasites to elude their hosts' immune system, and hence, an inflammatory response[4]–[9]. The strategies employed by viruses in the guise of cytokine and chemokine binding proteins, such as those directed against IFNγ, IL-18 and various chemokines have been fairly well documented[10]–[18].

Blood sucking parasites such as ticks can feed from several hours to several days, and thereby evade the host immune response. Anti-chemokine activity has been described in the extracts of tick salivary glands[5], [19], [20], and we have recently described the cloning and characterization of such chemokine binding proteins from a tick salivary gland cDNA library, which we have named Evasins[21], [22]. The first to be identified, Evasin-1, was shown to bind to a subpopulation of the chemokine family, contrasting with most of the known viral chemokine binding proteins which demonstrate broad selectivity profiles. Evasin-1 is a small 94-amino acid protein which binds CCL3/MIP-1α and CCL4/MIP-1β with very high affinity (0.16 and 0.81 nM, respectively), and also displays lower affinity binding (3.2 nM) to a closely related member of the CC chemokine family, CCL18/ PARC. This chemokine-binding protein does not share any relevant sequence or structural homology to any other known proteins, notably the viral chemokine-binding proteins, and moreover, is considerably smaller, being only 10 kDa compared to the viral proteins which range in size between 30–40 kDa.

Here we present the crystal structure of a complex between the chemokine CCL3 and the small tick-derived chemokine binding protein, Evasin-1. The interactions are totally different from those described for the viral chemokine binding proteins, and may lead to an understanding of an efficient way to selectively inhibit the chemokine system. The structure of the complex provides the structural framework for the exquisite selectivity demonstrated by Evasin-1, which displays a particularly high affinity for CCL3, but has only negligible affinity for the closely related chemokine CCL5, which shares the same receptors as CCL3. The binding activity of chimeric chemokine constructs, consisting of the amino terminal region preceding the CC motif of one of these chemokines, followed by the sequence of the other, suggest that the binding modality may follow a two-step process, with the amino terminus determining the selectivity.

## Results

### Architecture of Evasin-1

We have determined the crystal structure of both non-glycosylated (accession code: 3 fpr) and glycosylated Evasin-1 (accession code: 3 fpt), to 1.7 Å and 2.70 Å respectively. The structures are very similar, and the Cα can be superimposed with an rmsd of 0.97 Å, considering 83 of the 100 amino acid residues (segment aligned Asp8-Arg90). The structure of the non-glycosylated form of Evasin-1, which was determined at higher resolution, will be described below.

The crystal structure of the non-glycosylated Evasin-1 contains two molecules per asymmetric unit, monomer A (Asp5-Asp91) and monomer B (Gly10-Trp89). The terminal amino acid residues Asp5 and Asp91 for monomer A, as well as Trp89 for monomer B were modelled as alanines, as there is no unambiguous electron density for their side-chains. In both monomers, the extreme N-terminal and C-terminal regions, comprising the six-histidine tag, are flexible and were not seen in the electron density maps.

**Figure 1:**
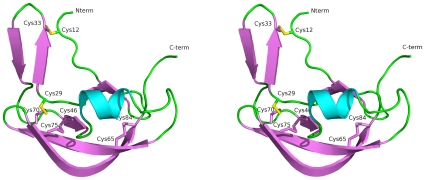
Overall structure of Evasin-1. A stereo view of the overall structure of the non-glycosylated form of Evasin-1 is presented.

The overall structure of the Evasin-1 molecule is boat shaped, with approximate dimensions of 35 Å × 20 Å × 13 Å. The largest dimension corresponds to the distance comprising the N-terminal region, which is exposed to the solvent and is involved in the chemokine binding, as shown later in the structure of the complex with CCL3. A stereo view of the overall structure of the non-glycosylated form of Evasin-1 is presented in Figure 1. This tertiary structure of Evasin-1 represents a new fold of the α/β type. A search against all the PDB structures deposited and currently available in the PDB did not identify any related structure, as will be discussed later in this paper.

**Figure 2:**
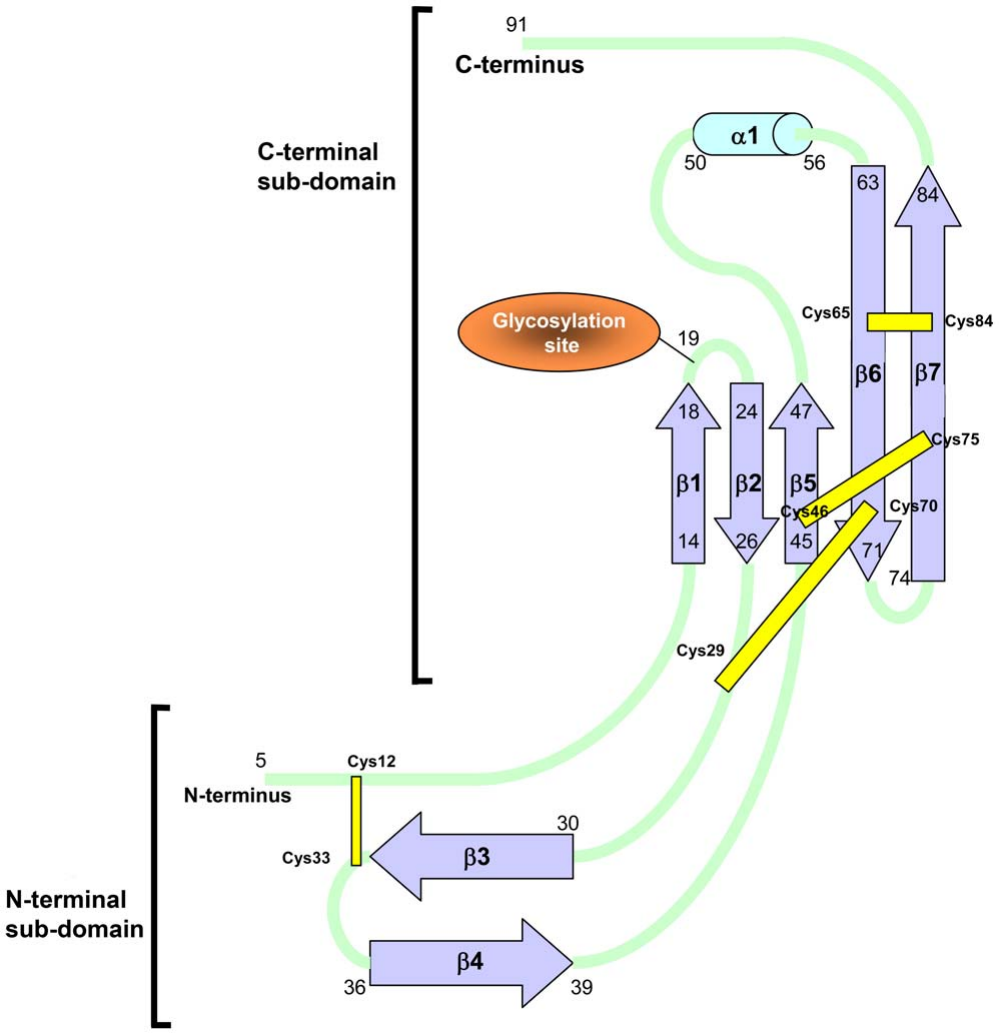
Secondary structure of Evasin-1. The secondary structure, disulfide bridges, and glycosylation sites of Evasin-1 are shown.

The Evasin-1 secondary structure is composed of seven beta strands forming three anti-parallel beta sheets, one short alpha helix, and contains four disulfide bridges (Figure 2). The four disulfide bridges consist of: Cys12-Cys33 connecting the N-terminal region of strand β1with β3; Cys29-Cys70 connecting the beginning of β3 with β6; Cys46-Cys75 connecting the strands β5 with β7 in the beta sheet β5-β6-β7 and finally Cys65-Cys84 connecting the other two strands β6 with β7 in the same beta sheet. The disulfide bridges are positioned along the interior of the protein, forming a central core, and undoubtedly conferring structural rigidity by stabilization of the protein core.

The overall architecture can be divided into two subdomains (Figure 2). The first subdomain (the N-terminal subdomain) is formed by two non-contiguous segments comprising the N-terminal residues Asp5-Pro13, and the anti-parallel beta-pleated sheet formed by β3 and β4 consisting on the segment from residues Lys30-Thr39. The beta sheet formed by β3 and β4 contains a beta-hairpin of type I'. A hydrogen bond network formed by Gln31-Glu38, Asp32-Gly35, Cys33-Gly35 and Cys33-Thr36 holds this beta sheet together.

The N-terminal segment is extended through the surface of the monomer and is covalently bound to the β3 strand via the disulfide bridge between Cys12-Cys33, which is exposed to solvent. The N-terminal region is anchored not only by the disulfide bridge, but also by an intricate hydrogen bond network that places the N-terminal segment in an exposed and favourable position for chemokine binding. This hydrogen bond network comprises the N-terminal region interactions with β4 through Gly11-Glu38, Cys12-Glu38, and with β3 through Pro13-Gln31. Residues Gln31 and Glu38 as well as Cys33, anchors the flexible N-terminal area. The Gln31 side-chain interacts with the carbonyl atom of Pro13 and also establishes main-chain and side-chain hydrogen bonds with Glu38, thereby stabilizing the beta sheet formed between β3 and β4.

The second, C-terminal, subdomain is also composed of two non-contiguous segments comprising residues Phe14-Cys29 and Ala40-Asp91. This subdomain is composed of five beta strands (β1-β2-β5-β6-β7), and one alpha helix (α1). This subdomain undoubtedly constitutes the central core of the protein, as evidenced by the lower B-factors observed in this extended β-sheet. The beta strands β1 and β2 form an anti-parallel beta sheet containing a beta turn of type 1. The beta strands β5, β6 and β7 also form a twisted anti-parallel beta sheet. The two beta sheets formed by β1-β2 and β5-β6-β7 interact together to form the twisted beta-barrel (β1-β2-β5-β6-β7-β1), which is pointing to the alpha helix on the top. β1 and β2 are bridged through the main-chain hydrogen bond interactions between Ala17 and Thr25. Asn19 plays an important function by holding the beta turn of type I, due to the six hydrogen bonds that it makes with neighbouring residues Thr21, Gly22, Tyr23. Remarkably the ND2 atom is not involved in these interactions, which is consistent with it being a glycosylation site, an observation that is confirmed in the glycosylated structure of Evasin-1, as described later.

The α1 helix is positioned opposite of the twisted beta barrel vortex subdomain (as depicted in Figure 1). This helix is placed in a solvent exposed area and is held to the vortex through three hydrogen bonds, one between the start of the helix and the end of β2 Gly50-Val27, one between the middle of the helix and the C-terminal Arg55-Arg86, and one at the end of the helix and the C-terminal Met58-Arg86. The C-terminal loop points to the solvent, presenting the aromatic residue Trp89 totally exposed and located in a flexible region with a Cα B-factor = 50.0 Å^2^, much higher than the average B-factor  = 34.8 Å^2^ for the whole protein chain of monomer A (B-factor for the Cα of monomer A  = 32.8 Å^2^, calculated with BAVERAGE from CCP4, 1994).

### Glycosylated Form of Evasin-1

Three glycosylation sites were initially predicted by primary sequence analysis through a PROSITE search used for pattern identification (http://www.expasy.org/prosite)[23]. A typical N-glycosylation consensus pattern Asn-Xxx-Thr[24] was found for Asn19-Lys20-Thr21, Asn34-Gly35-Thr36 and Asn42-Gly43-Thr44, which are located in external loops, not involved in chemokine binding (see Figure 2).

Crystals of the glycosylated tick chemokine binding protein produced in Sf9 insect cells using the baculovirus expression system were obtained as described in the Methods section. The structure was solved by molecular replacement using the previously determined structure of the non-glycosylated form of Evasin-1. The unit cell contains 3 molecules per asymmetric unit: monomer A (Asp8-His95), monomer B (Gly11-Trp89) and monomer C (Asp8-Trp89). The corresponding solvent content of 67%[25] and the glycosylation content may explain the 2.70 Å diffraction limit of the crystals. The three crystallographic distinct molecules are very similar with a rmsd for Cα atoms superposition of 0.68 Å for the superposition of monomers A and B, 0.77 Å for monomers B and C, and 0.95 Å for monomers B and C, with the greatest differences being observed for the residues at the termini. Monomer A had the lowest overall B-factor (53.9A^3^) compared to the other two monomers, had the largest stretch of visible amino acids (8 to 95), and will thus be used as the reference glycosylated structure in the discussion below.

Residue Asn19 was the only one of the three predicted glycosylation sites, which displayed clear electron density for a sugar moiety. Only one saccharide unit, built as an N-acetyl D-glucosamine, could be successfully modelled into all three monomers of Evasin1-glycosylated on residue Asn19. There was some unclear electron density visible for the next monosaccharide in monomer A, and it was thus not modelled. Asn19 is at the C-terminal end of the 1^st^ β-strand (β1) and is well exposed to solvent. There is a clear hydrogen bond interaction between the acetamide group of the glucosamine and the side chain of Thr22.

### Architecture of the Complex between Evasin-1 and CCL3

**Figure 3:**
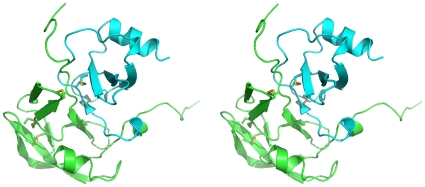
Stereo diagram of the complex between Evasin-1 and CCL3. The Evasin-1 is colored in cyan and CCL3 in green.

In order to understand the structural basis of the chemokine binding mechanism by Evasin-1, we have crystallized the complex (accession code: 3 fpu) between Evasin-1 and a variant of the human chemokine CCL3 (Figure 3). The crystal structure was refined up to 1.9 Å resolution, and it helps to clarify the structural features of the binding mode of a new class of chemokine binding proteins. One single complex of Evasin-1 and CCL3 crystallised in the asymmetric unit.

One monomer of Evasin-1 binds one monomer of CCL3 displaying a 1∶1 stoichiometry. Upon complex formation, the N-terminal region of CCL3 interacts with both the N-terminal and C-terminal regions of Evasin-1. These extremities, which are not completely visible in the isolated crystal structures due to their intrinsic disorder and flexibility, become more rigid due to a network of interactions between the two proteins when the complex is formed. As a consequence, some additional secondary structure elements become visible. In the refined structure of the complex, it is possible to identify the entire Evasin-1 molecule in the electron density, as well as the CCL3 structure with the exception of the last 2 residues of the C-terminus. The crystal structure reveals the C-terminal polyhistidine tag, and includes two metal ions, modelled as Ni^2+^ atom (believed to be leached from the IMAC resin during purification) and which play an important role in establishing the crystal lattice.

The overall topologies of both Evasin-1 and CCL3 in the complex are similar to the isolated crystallographic structures. The Cα superposition of the isolated Evasin-1 (monomer A) with the respective structure in the complex gives an overall rmsd of 1.0 Å. The Cα superposition of the crystallographic structure of the CCL3 (J. Dias, unpublished results) and the respective structure of CCL3 in the complex also gives an overall rmsd of 1.0 Å, considering only the C-terminal 50 amino acid residues.

**Figure 4:**
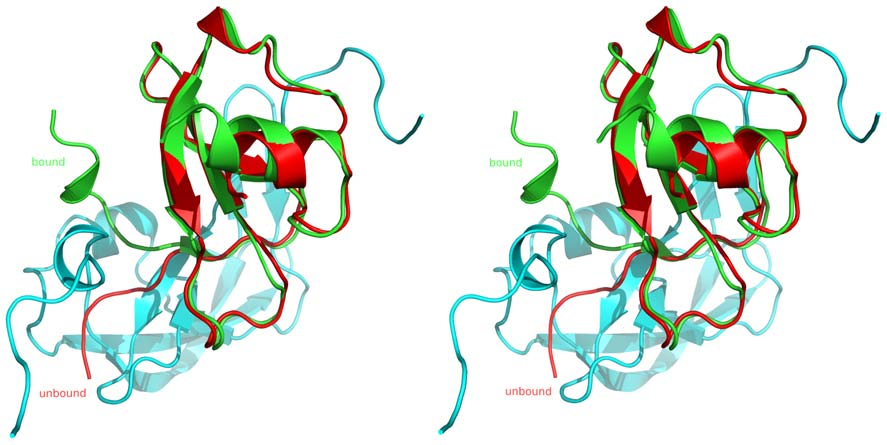
Stereo diagram of the comparison of unbound CCL3 with CCL3 bound to Evasin-1. The unbound form of CCL3 is shown in red, the bound form displayed in green.

The complex formation induces the stabilization of the entire N-terminal and C-terminal areas of Evasin-1 and of the N-terminal segment of CCL3, which becomes visible in the electron density maps, showing the rearrangement of both N-terminal ends upon binding. Due to interactions in the complex, the CCL3 N-terminal region (residues Ser2-Thr10) moves by almost 90 degrees with 5AlaB moving circa 20 Å (Figure 4). This difference in the structure of the free and Evasin-1 bound structure is due essentially to rotation around the Pro8-Thr9 and Thr9-Thr10 peptide bonds. Three additional secondary structure elements are formed upon binding: the α2 3-10 helix at the C-terminal of Evasin-1; and the α0 3-10 helix and β0 strand at the N-terminal region of CCL3. The additional CCL3 β0 strand interacts with the Evasin-1 β1 forming an antiparallel beta-sheet, which extends to Evasin-1 β2. The newly formed CCL3α0 (Ala4A-Asp6A) interacts with the newly formed Evasin-1 α2 3-10 suggesting that this stabilization occurs in a cooperative manner.

**Figure 5:**
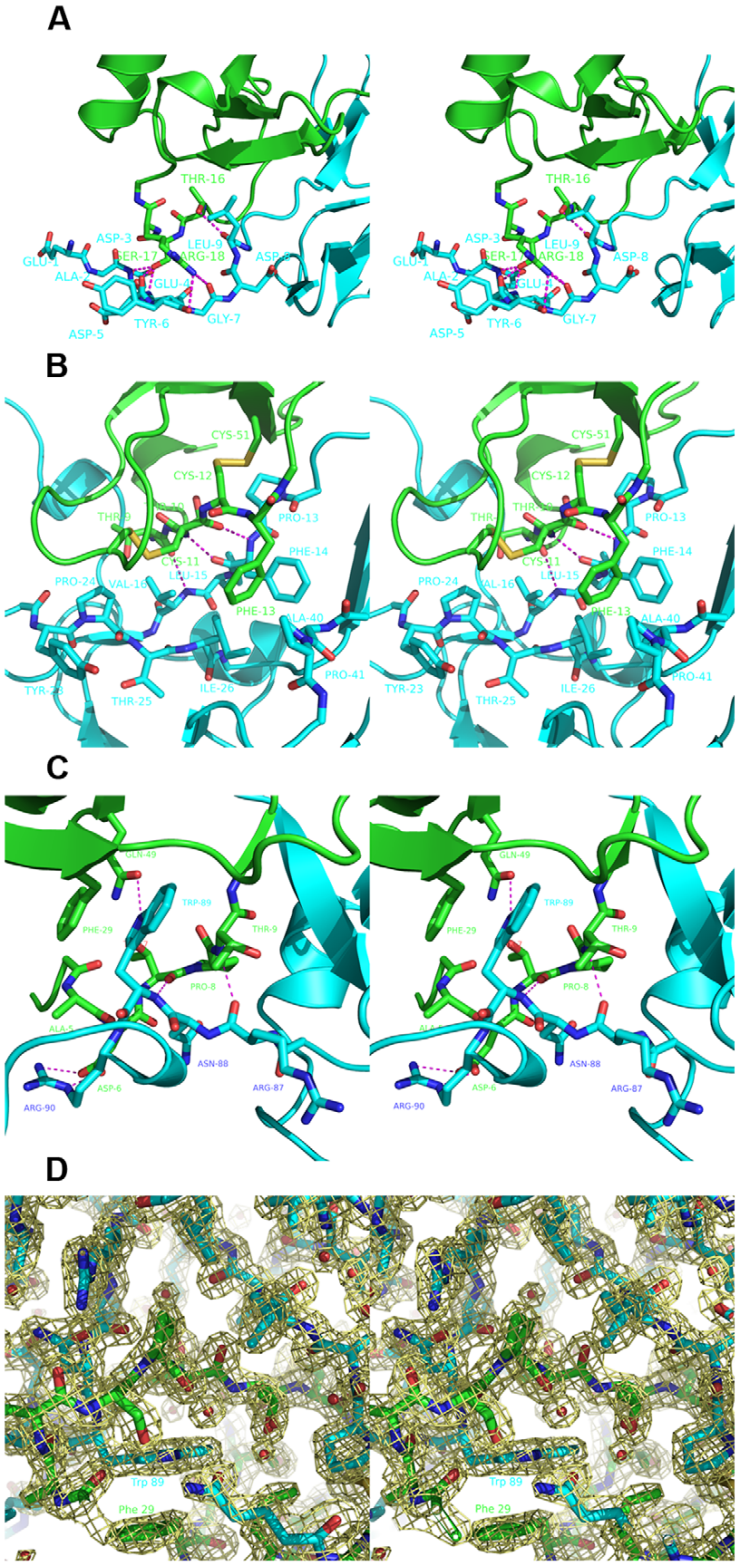
Close-up of the interactions between Evasin-1 and CCL3. The Evasin-1 is colored in cyan and CCL3 in green. (A) Interaction between the Thr16-Ser17-Arg18 loop of CCL3 with Evasin-1 (B) Interaction between Phe13 of CCL3 and Phe14 of Evasin-1 (C) Interaction between Phe29 of CCL3 and Trp89 of Evasin-1 (D) 2Fo-fc electron density map, contoured at 1.5σ of the interaction of the N-terminal region of CCL3 with Evasin-1.

Due to stabilization of the structures promoted by the interaction within the complex, and due to the crystal packing, the C-terminal of Evasin-1 becomes visible and the 3–10 helix formed by Trp89A-Lys92A is revealed. The Trp89A is perfectly visible (see Figure 5c) in the electron density map for the complex, and the interacting residue Phe29B goes through a rotamer change in order to accommodate this hydrophobic interaction.

### Domain Interactions at the Interface of the Complex

The total buried surface area at the interface of the complex is 2650.58 Å^2^ as determined by CNX[26] or 2923.5 Å^2^ as determined by the CCP4 program AREAIMOL[27]). The shape complementarity of the Evasin-1 /CCL3 interface was calculated with SC from CCP4 to be Sc = 0.76 using a 1.7 Å probe sphere radius[28].

The chemokine-binding region of Evasin-1 is an extended region comprising both the N-terminal and C-terminal regions, which embrace the chemokine. The interface of the complex was calculated with CNX and LIGPLOT/HBPLUS and followed by visual inspection and is summarized in Table S1). This interface comprises 35 residues from the Evasin-1 and 27 residues of CCL3. The hydrogen bond network at the interface of the complex is composed of 25 hydrogen bonds, consisting in the interaction of 18 residues from the Evasin-1 and 12 residues of CCL3 (CONTACT from CCP4[29]).

Three specific residues of CCL3, Thr16B-Ser17B-Arg18B, are targeted by the N-terminal region of Evasin-1, through 7 hydrogen bonds (Figure 5a). The main-chain of the N-terminal residues Asp3A-Leu9A of Evasin-1 interacts with the side-chain of this unique segment of CCL3. This interaction is very specific since it targets mainly the side-chains of this Thr16B-Ser17B-Arg18B motif, which is unique to CCL3. A sequence alignment of chemokines revealed a maximum of two identical residues, but never the exact three-residue motif. The region of CCL3 may thus be an important determinant of the unusual selectivity of Evasin-1.

The N-terminal β1 strand of Evasin-1 interacts with the Thr9B-Cys11B N-terminal region of CCL3 through 3 hydrogen bonds, inducing a short anti-parallel beta strand conformation upon complex formation, assigned as strand β0 and formed by Thr10B-Cys11B. The N-terminal contacts include several hydrophobic interactions, one of which is an important edge-to-face π-π interaction between Phe14A from Evasin-1 and Phe13B from CCL3. This hydrophobic interaction is strengthened by a main-chain hydrogen bond interaction between Evasin-1 Phe14A and CCL3 Cys11B, connecting both N-terminal regions and holding Phe14A in the appropriate environment (Figure 5b). Surrounding the Phe14A-Phe13B interaction, there is a hydrophobic patch in Evasin-1 formed by residues: Pro13A, Phe14A Tyr23A, Pro24A, Ile26A, Ala40A, Pro41A, while the corresponding CCL3 adjacent “hydrophobic” region is formed by the disulfide bridges between Cys11B-Cys35B and Cys12B-Cys51B, which lie in the vicinity (less than 4 Å) of Pro24A and Pro13A, respectively.

The C-terminal of Evasin-1 embraces the N-terminal part of CCL3 due to an important network of hydrogen bonds and comprising a very important hydrophobic stacking interaction between the Trp89A from Evasin-1 and Phe29B of CCL3 (see Figure 5c). The aromatic side chains of Trp89A from Evasin-1, and Phe29B CCL3 are arranged parallel but slightly off centre, as observed in Figure 5c. Trp89A NE1 of Evasin-1 also interacts with Gln49B OE1 from CCL3, which anchors the Trp89A in a favourable position for the aromatic stacking with Phe29B from CCL3. The N-terminal domain of CCL3 plays a major role in the interaction with Evasin-1 by forming a lid to the pocket that anchors Trp89A from Evasin-1. The segment after the N-terminal helix of CCL3 composed by the residues: Ala5B-Asp6B-Thr7B-Pro8B-Thr9B-Thr10B encloses the Trp89A (Evasin-1) – Phe29B (CCL3) interaction. CCL3 Pro8B fits in the pocket of Evasin-1 defined by: strands β1 (Leu15A) and β7 (Arg86A, Asn88A, Trp89A), with some side-chains from helix α1 (Leu54A, Arg55A), interacting with Gln49B via a bridging water, contributing to the positioning the hydrogen bond Gln49B OE1-NE1 Trp89A Evasin-1.

### Electrostatic Complementarity

**Figure 6:**
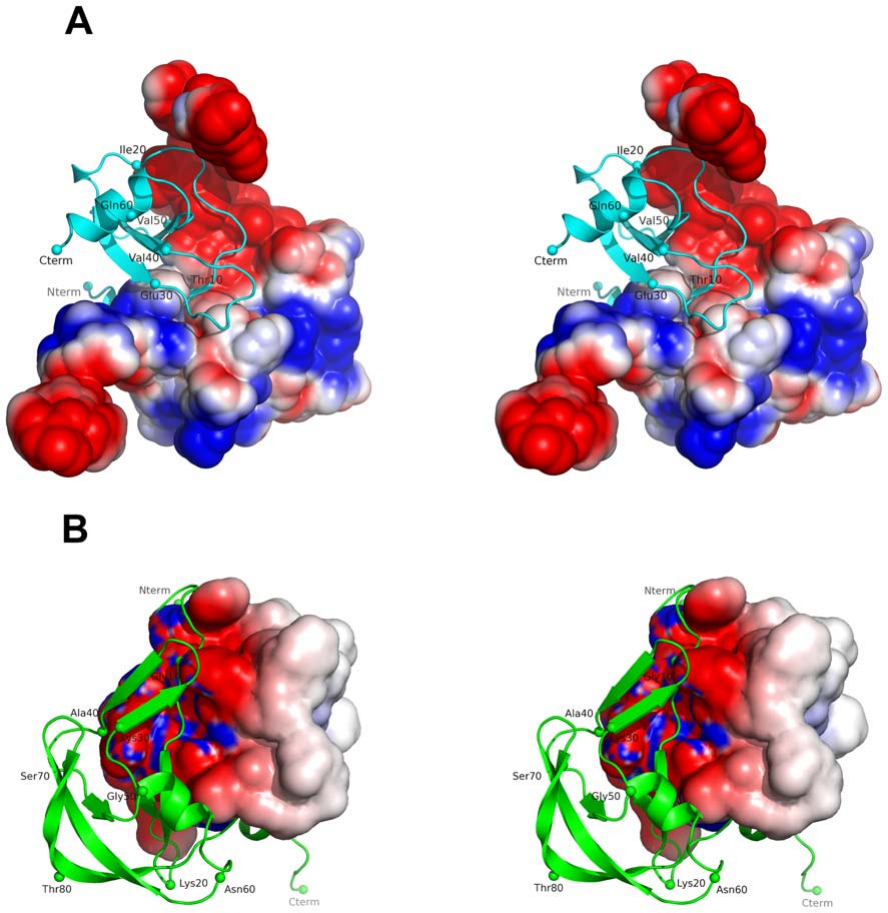
Electrostatic surface complementarity between Evasin-1 and CCL3. (A) The CCL3 molecule is displayed as a cyan-colored ribbon, while the Evasin-1 is displayed as a molecular surface colored by surface electrostatic potential. (B) The complex in (A) is rotated 180° along a central vertical axis and the Evasin-1 is displayed as a green ribbon and the CCL3 molecule as a molecular surface colored by surface electrostatic potential.

The relatively small difference in the isoelectric points (pI) of Evasin-1 (pI = 6.0) and CCL3 (pI = 4.9), compared to more basic chemokines like RANTES (pI = 9.3), may suggest that hydrophobic interactions are the main driving forces for the complex formation between Evasin-1 and CCL3. Nevertheless, and despite the relatively small difference in the overall protein charge, there is a remarkable electrostatic surface complementarity at the contact interface of the complex that enhances the complex interactions (Figure 6). A remarkable feature at the interface of the complex is the electrostatic complementarity observed surrounding the aromatic stacking interaction between Trp89A-Phe29B, in which the Phe29B belonging to CCL3 is buried in a negatively charged pocket due to the presence of Glu30B and to the proximity of Asp6B from CCL3 N-terminal region. On the other hand, the Trp89A is enclosed in the positive C-terminal of Evasin-1. The Trp89A-Phe29B interaction is surrounded by an electrostatic ring that helps to orient both partner molecules, guiding the protein docking through an electrostatic field.

## Discussion

### Novelty of the Evasin-1 Structure

Several aspects of the structure of the complex between Evasin-1 and CCL3 are striking. A preliminary analysis of the amino acid sequences of Evasin-1 by BLAST and PHI-BLAST had revealed the absence of any protein with a similar amino acid sequence. We have recently identified two other chemokine binding proteins from tick saliva, Evasin-3, a highly selective CXC chemokine binding protein and Evasin-4, a CC chemokine binding protein, cloned by cross-linking to CCL5 and CCL11/Eotaxin[30]. Similarly, these proteins have unique sequences, but Evasin-4 has the same pattern of cysteine residues as Evasin-1, suggesting that these two proteins will share a common fold. The resolution of the structures of Evasin-1 and Evasin-3[30] has confirmed the novelty of the structure of these proteins. An analysis of the secondary structure pattern of Evasin-1 depicted in Figure 2 has revealed no significant similarities with other known proteins.

### Comparison of the Mode of Binding of Evasin-1 Compared to That of Other Chemokine Binding Proteins

**Figure 7:**
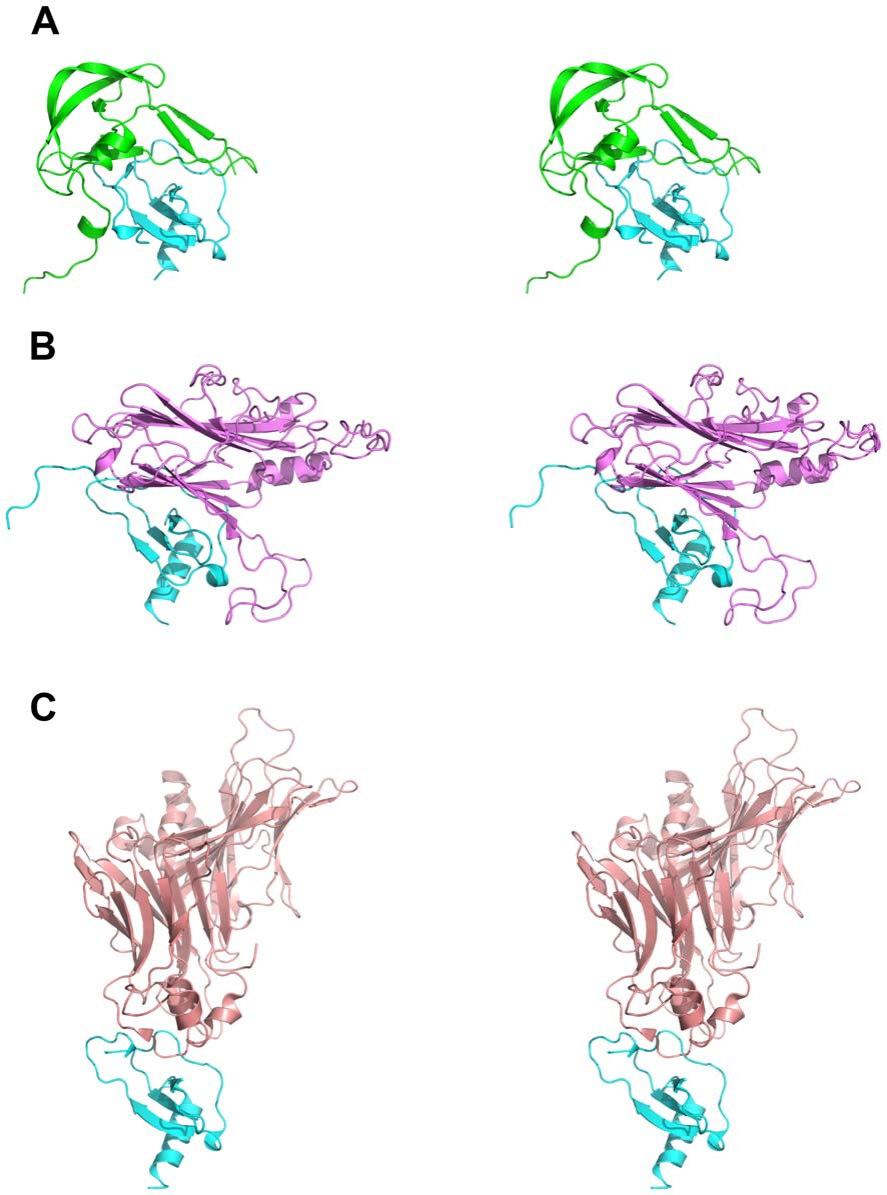
Stereo diagram comparing the structure of the complexes of CC chemokines with different CC chemokine binding proteins. (A) Ribbon diagram of the complex of CCL3 with Evasin-1. CCL3 is displayed in cyan and Evasin-1 in green. (B) Ribbon diagram of the complex of CCL4 with vCCI. CCL4 is displayed in cyan and vCCI in violet, (C) Ribbon diagram of the complex of CCL2 with M3 decoy receptor. CCL4 in cyan and the M3 decoy receptor in mauve.

Herpes or poxviruses express most soluble chemokine-binding proteins identified or characterized to date. These proteins are believed to disrupt chemokine interactions with host cell receptors or glycosaminoglycans, the latter interaction being required for their immobilization in the circulation. Among the best characterized of these viral chemokine-binding proteins are the leporipoxvirus and orthopoxvirus encoded viral CC chemokine inhibitor (vCCI) family, which display selectivity towards CC chemokines[31]. These proteins have been shown to not only have potent anti-chemokine activity *in vitro*, but also to display anti-inflammatory activity *in vivo* [32]. The binding mode of the small tick-derived Evasin-1 to CCL3 can be compared to that of a much larger poxvirus-encoded CC chemokine-binding protein to CCL4, a very close homologue of CCL3, since the NMR solution structure of the complex has been determined (pdb entry 2 ffk and 2 fin)[33], [34]. The CCL3 and CCL4 sequences in the complex structures are very similar, with over 60% identity and 70% similarity, and the structures are very similar, as can be expected, with the exception of the extreme N-termini of CCL3 and CCL4, with the main chains displaying an important difference in position between residues 1 and 10. The N-terminus of CCL4 in the vCCI:CCL4 complex closely resembles that of the uncomplexed crystal structure of CCL3 (J. Dias, unpublished results), and the important movement observed around the peptide bond between Pro8-Thr9 and Thr9-Thr10 of CCL3 in the Evasin-1:CCL3 complex remains unique. The chemokine-binding proteins are very different, however, in structure (see Figure 7 ab), size (vCCI is 26 kDa compared to 10.4 kDa for Evasin-1), and selectivity[31], but they both interact with a similar region of the chemokine ligand (see Figure 7c). The complexes are thus also very different in size, shape, and also in buried surface area (2650 Å^2^ for the Evasin-1:CCL3 and only 1980 Å^2^ for the much larger vCCI:CCL4 complex). Whilst the region of the chemokine that interacts with the chemokine-binding protein is the same, many of the specific amino acids involved in the interactions are not. The 6 important hydrogen bonding interactions between CCL3 Thr16-Ser17-Arg18 and Evasin-1 are not reproduced in the vCCI:CCL4 complex, despite the close sequence similarity of CCL4 with CCL3 (identical except for residue 17, which is an Ala instead of a Ser). Surprisingly, vCCI does have complementary residues in the vicinity of CCL4, but the hydrogen bonds would appear to be rather weak (3.2 Å between Asp141 Oδ2 of vCCI and the Nε of Arg18 of CCL4 and between the Oε1 of Glu143 and the Oγ of Thr16 of CCL4). The β-strand at residues 10-11 of CCL3 induced by binding of Evasin-1 is not present in the vCCI:CCL4 complex, due to the important differences in the structure of the N-terminal regions of the two chemokines. The interesting edge-to-face π-π interaction observed between Phe13 of CCL3 and Phe14 of Evasin-1 is not present in the vCCI:CCL4 complex, where the corresponding hydrophobic pocket is composed, for the most part, of aliphatic hydrophobic side chains. This is interesting because of the role attributed to the side chain of Phe13 of CCL4 in receptor binding[35]. The important stacking interaction between Phe29 of CCL3 and Trp89 of Evasin-1 is not present in the vCCI:CCL4 complex, where the Tyr14 side chain of CCL4 is found pointing into solution, and making no contact with vCCI. It would thus appear that the interactions between the chemokines with Evasin-1 or vCCI are not conserved.

The other class of virally-encoded chemokine binding proteins for which a complex structure has been determined is the M3 decoy receptor from murine herpesvirus-68, whose structure with, and without CCL2, a member of the CC chemokine family has been determined[36]. The structure of this chemokine-binding protein is quite different from that of either Evasin-1 or vCCI, as shown in Figure 7c. The M3 protein is even larger than vCCI (42 kDa), and appears to be a dimer in solution, the dimer binding two molecules of chemokine to form a complex with a stoichiometry of 2∶2. The buried solvent accessible surface area of the complex of approximately 2600 Å^2^ is similar to that observed for the Evasin-1:CCL3 complex.

The chemokine that was initially used to determine the structure of this complex was an obligate monomer version of CCL2, with the Pro8 mutated to Ala; but the solution of the structure of the complex containing the wild type CCL2 showed no differences [37]. In both structures, as well as that of the complex with another chemokine, XCL1, the N-terminal areas of the chemokines were not observed in the crystal structure. The M3 proteins make no interaction with the Thr16-Ser17-Arg18 region that is so important in the case of the CCL3-Evasin-1 complex, and a tyrosine residue in CCL2 replaces the Phe13 residue in CCL3. It is interesting to note that an edge-to-face π-π interaction would appear to take place between this Tyr13 and Tyr266 of M3, reinforcing the possibility that this residue plays an important role in chemokine-receptor interaction. The important stacking interaction observed between CCL3 Phe29 and Evasin-1 Trp89 is also not observed in the M3:CCL2 complex, since this part of the chemokine is not in close proximity to the M3 protein.

The examination of these three complexes, all of which contain similar chemokines of the CC sub-class, highlights the amazingly different ways in which nature has evolved binding modes to neutralize chemokines. The most striking difference amongst the chemokine binding proteins is, of course, their different sizes, and binding modes. It is interesting to note that they all contain folds that are unrelated to known protein folds. It would appear that the binding to chemokine molecules has requirements that cannot be met by the known classical protein folds commonly found in higher species.

### Structural Basis for the Selectivity of Evasin-1

**Figure 8:**
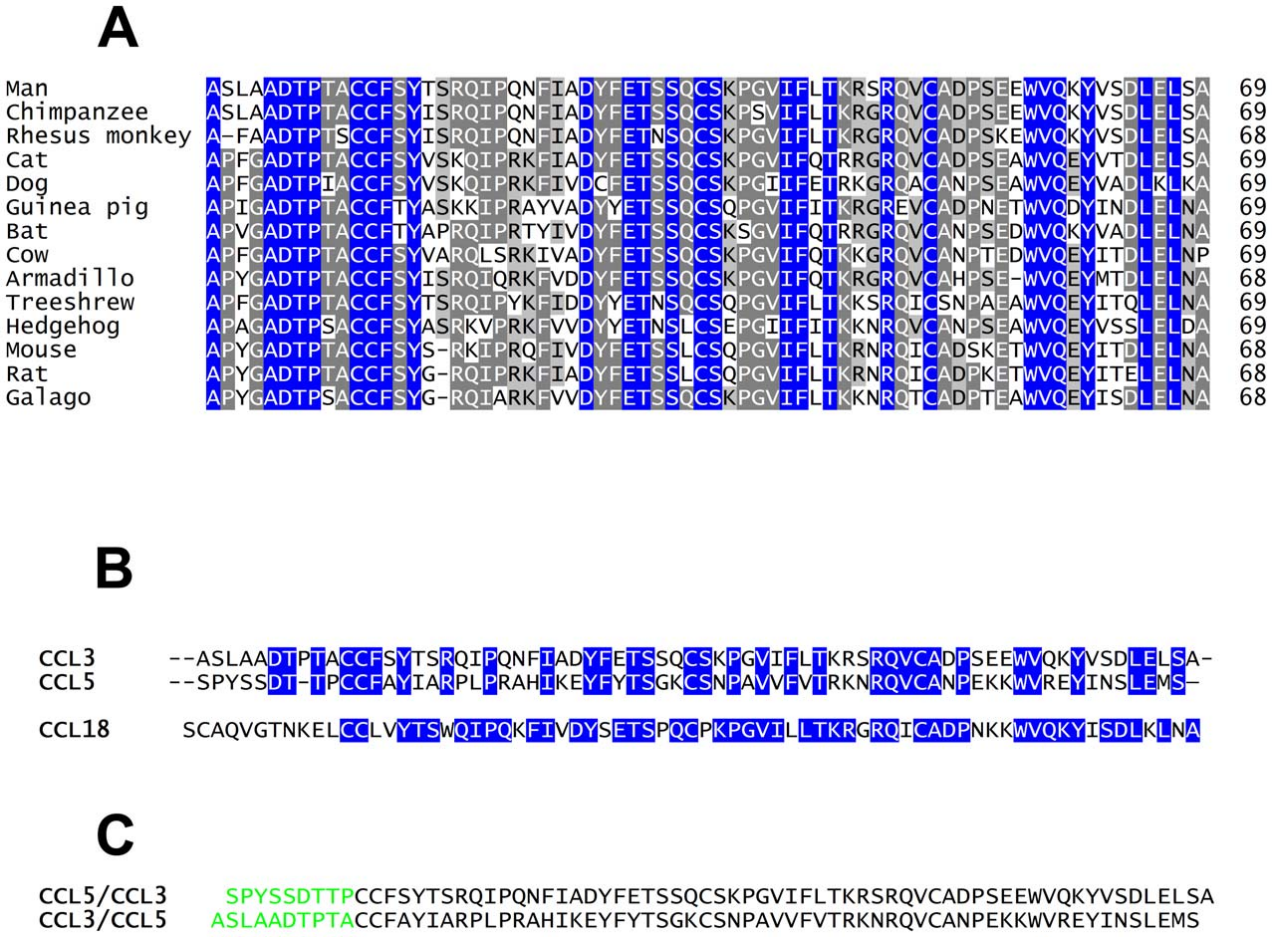
Amino acid sequence alignments. (A) Alignment of mammalian CCL3 sequences. Fully conserved residues are background colored in blue, highly conserved (>80% identity amongst the species shown) in dark grey, and poorly conserved (>60% identity) in light grey. (B) Alignment of chemokines towards CCL3. The blue background identifies amino acids that are identical to CCL3. (C) Amino acid sequences of the chemokine chimera.

The tick *Rhipicephalus sanguineus*, also known as the common brown dog tick, is usually found feeding on dogs but is known to infest other mammalian species. In view of this broad host range of it is likely that Evasin-1 is also able to inhibit CCL3 from other species. We have shown that Evasin-1 is indeed able to bind to mouse CCL3 with a comparable affinity to that measured for human CCL3, but binding to CCL3 from other species has not been tested. It was therefore interesting to determine how similar the sequence of CCL3 is between species. The amino acid sequence of CCL3 is remarkably conserved in mammals (Figure 8a). For example, the Phe29 residue, which makes the stacking interaction with Trp89 of Evasin-1, is conserved in all mammalian species. The Thr16-Ser17-Arg18 region of CCL3 is less well conserved between species. The Arg18 residue is replaced by lysine in several mammalian species (notably dog), but this is a conservative substitution that should not affect the selectivity of Evasin-1, though it may affect the binding affinity. The Thr16 residue is far less well conserved in mammals, being replaced by substantially different amino acids (Ile, Val, Ala and Tyr). In fact, this is one of the least well-conserved residues in the mammalian CCL3 family, and yet would appear to play an important in the interaction between the two proteins, notably forming a hydrogen bond with the peptide oxygen of Leu9 of Evasin-1. Ser17 is also not particularly well conserved throughout the CCL3 family either, despite its side chain forming several interactions with Evasin-1. This loop cannot be responsible for the high selectivity displayed by Evasin-1.

**Figure 9:**
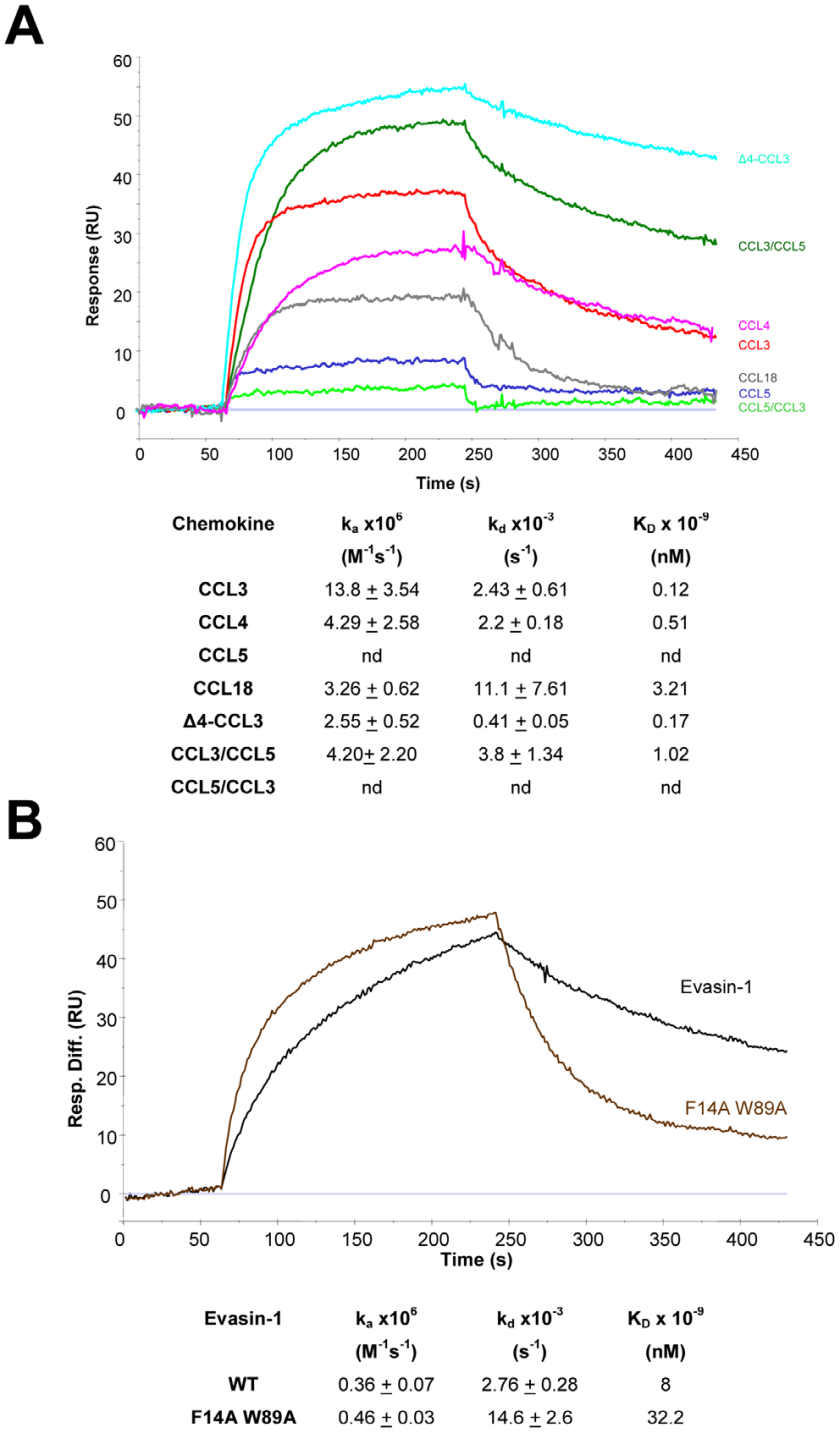
The N terminus of CCL3 is involved in the selectivity of Evasin-1 binding. Upper panels: sensograms obtained for binding experiments, lower panels: kinetic parameters relative to binding experiments. A) Chemokine binding to immobilized Evasin-1. Sensogram corresponding to CCL3/CCL5 (green) shows similar binding properties to CCL3 (red) and Δ4CCL3 (cyan); CCL5/CCL3 (light green), and CCL5 (blue) are unable to bind, as is CXCL8. nd  =  not determined; the affinity of the chemokine was too low for accurate measurement. B) Evasin-1 WT (Black) or F14A W89A (brown) to immobilized CCL3.

We believed that the solution of the structure of the complex of Evasin-1 with CCL3 would reveal the reason for the surprising selectivity of the former for the latter. We were therefore surprised by the fact that modelling the sequence of CCL5 into the structure of the complexed CCL3 suggested that Evasin-1 should bind this chemokine, contrary to the experimental results, in which we were never able to demonstrate CCL5 binding by cross-linking or by surface plasmon resonance (Fig. 9a). CCL5 has the two hydrophobic residues in the correct position that could bind to F14 and W89 of Evasin-1, but no binding to CCL5 was observed by the different assays we used. However our initial conclusion that the two “π-π” interactions were the major binding forces appeared to be incorrect, since the mutation of these hydrophobic residues to Ala had only a small impact on the affinity for CCL3 (Fig. 9b). It should be noted that the affinity of the WT and the F14AW89A Evasin-1 proteins was measured on immobilized chemokine for a direct comparison, which consistently results in significantly lower affinities (results not shown). Mutation of these two residues had no impact on k_on_, but did affect k_off_, suggesting that they play a role in the stability of the complex, rather than in the selectivity of the binding protein for CCL3.

The next surprising finding was that Evasin-1 was capable of binding a CCL3/CCL5 chimera (see Figure 9), in which the first 10 amino acids at the N- terminus were from CCL3, and the subsequent 59 are from CCL5, with an affinity similar to the wild-type CCL3. This was unexpected since the full-length CCL5 did not show any affinity towards Evasin-1. Furthermore, the opposite chimera, CCL5/CCL3, did not bind at all, thus illustrating the importance of the amino terminal region. Moreover, the fact that the affinity is unchanged for the Δ4 form of CCL3, the truncated form found in biological fluids[38] indicates that the selectivity resides in the 6 residues immediately preceding the CC motif. These 6 amino acids are, not surprisingly, highly conserved amongst the CCL3 of different species (Figure 8a). Of the six amino acids immediately upstream of the CC motif, ^4^ADTPTA^10^, Asp6, Thr7 and Pro8 are extremely highly conserved in all mammalian species and play an immediately identifiable role in the structure of the complex; the side chain of Asp6 forming important hydrogen bond interactions with the side chain of Arg90 from Evasin-1, the main chain carbonyl of Thr7 by interacting with the main chain nitrogen of Trp90 of Evasin-1, and it would thus appear that Evasin-1 recognizes its target chemokine through this sequence, and subsequently binds the rest of the protein.

The major difference in the N-terminal amino acid sequence of CCL5 when compared to CCL3 is the lack of a residue equivalent to the Pro8 in CCL3. An insertion has to be made in the alignment of the two sequences, since the residues Asp6 and Thr7 are conserved, and both make important contributions to the complex interface. The lack of this residue could therefore be responsible for the lack of affinity of Evasin-1 for CCL5. The K_a_ for the CCL3/CCl5 chimera is reduced 3-fold compared to CCL3, while the K_d_ is not particularly affected, suggesting that CCL5 may play a negative role in the binding event, whilst not unduly influencing the stability of the complex. It is thus probable that the preferred ligand for Evasin-1 is actually CCL3 in which the amino terminus serves as the “address” but that Evasin-1 is capable of binding closely related family members albeit with a slower on-rate, since the body of the chemokine will compensate for the remainder of the binding interactions. It should be noted that these hypotheses are drawn in an attempt to explain the exquisite and perplexing selectivity of this binding protein and remain to be substantiated with experimental evidence. However, this current hypothesis is not supported by the affinity of Evasin-1 towards CCL18, since the sequence of the N-terminus of this chemokine bears no resemblance to that of CCL3. However, CCL18 was apparently derived through gene duplication of the CCL3 gene[39], and whilst the structure of this protein is unknown, there may be other structural features in CCL18 that explain its affinity for Evasin-1.

There is evidence that ticks produce different chemokine binding proteins at different times during the feeding cycle, in contrast to the more non-selective strategy employed by viruses. The reason for this is open to speculation, but may be associated with the fact that ticks harbour a large genome (>1×10^9^ bases) with the potential of encoding multiple CK binding proteins, unlike viruses, which are limited by much smaller genomes. Nevertheless it is of interest that although both Evasin-1 and Evasin-3, which are distinct in terms of structure and sequence, are both small proteins (<10 kDa) similar in size to protein binding scaffolds such as single chain camelid antibodies, named nanobodies[40] or scaffolds such as ankyrins[41], [42] etc, that are increasingly being developed as protein therapeutics. Parasites such as ticks have apparently developed this strategy before mankind, and the novelty of this chemokine-binding fold may reveal features for neutralization of important immunomodulatory proteins such as chemokines that could help us design improved biological therapeutics. We are currently investigating the importance of the different residues in the interaction between Evasin-1 and CCL3, and the role this plays in the selectivity of Evasin-1, by site-directed mutagenesis of both partners. Information gained from such studies may enable the design of Evasins with defined chemokine binding specificity that could be therapeutically useful in inflammatory and infectious diseases, and cancer.

## Material and Methods

### Baculovirus Production of Evasin-1

Evasin-1 was expressed in insect cells and purified by chromatography as described elsewhere[43]. We amplified and subcloned the full-length Evasin-1 cDNA, including its signal peptide for secretion, into a pDEST8 expression vector (Invitrogen) with a 6-histidine tag sequence at the COOH-terminus. *Spodoptera frugiperda* (Sf9) cells were transfected and the recombinant virus was amplified using standard methods. Evasin-1 was expressed using the baculovirus system in insect cells (Bac-to-Bac, Life technologies/Invitrogen). Baculovirus harbouring full length C-terminal His-tagged Evasin-1 ORF were then used to infect either Sf9 insect cells in SF900 II medium (Invitrogen), or *Trichoplusia ni* (High Five) (Tn5) insect cells in Ex-cell 405 medium (JRH Biosciences), at 27°C (the yield obtained with Sf9 cells was 10 times lower, so Tn5 cell expression was used for scale-up). For large-scale production, several 2-L flasks of Tn5 cells were grown to a density of 2.0×10^6^ cells per mL, which were then infected with recombinant virus at a multiplicity of infection (MOI) of 10.0. Cultures were allowed to grow for 64 hours post-infection, before the cells were harvested by centrifugation. The mature protein, with its signal peptide cleaved, is secreted into the supernatant. The supernatant was filtered and the volume of the supernatant was reduced to 500 ml by tangential flow concentration and dialyzed against 50 mM phosphate buffer pH 7.5, 0.3 M NaCl, and 5 mM imidazole, and was immediately purified.

In order to obtain non-glycosylated protein, Evasin-1 was expressed in the presence of tunicamycin, a known inhibitor of N-linked glycosylation[44]. Tunicamycin (0.2 µg/ml) was added to the culture media immediately before infection, and the expression of the protein was followed by Western-blot. The non-glycosylated protein was purified by methods identical to those used for the glycosylated form.

### Evasin-1 Protein Purification

Both the glycosylated and non-glycosylated proteins were soluble and were purified to homogeneity in three steps, using a similar protocol. The his-tagged protein was captured initially using an affinity column (Ni-NTA), which was followed by anionic exchange (Q resource) and finally gel filtration (Superdex 100) on a Pharmacia FPLC system. The final sample was concentrated up to 10 mg/ml, in the final buffer (100 mM Tris-HCl, 100 mM NaCl, pH 7.5) ready for crystallization purposes. The homogeneity of the sample was followed through all steps of the purification by SDS-PAGE.

The glycosylated protein was heterogeneous; presenting a smeared band in SDS-PAGE, corresponding to different species of glycosylated protein, which was confirmed by isoelectric focusing.

The non-glycosylated protein showed a single band on SDS-PAGE, but the isoelectric focusing revealed two major bands, which were separated in the anionic exchange (Q-Resource) step. The two fractions were analysed by N-terminal sequencing and mass spectrometry, which revealed that they had molecular masses of 11286 Da and 11362 Da, respectively. The first fraction (pI = 6.0), which corresponded to the mass of the non-glycosylated protein, yielded crystals, while the second fraction (pI = 5.8) with an extra 76 Da did not crystallize.

### Crystallization and Structure Determination of the Non-Glycosylated Evasin-1

Glycosylated Evasin-1 was submitted to deglycosylation studies using different endoglycosidases (Endoglycosydase Hf, PNGaseF – Peptide-N-Glycosydase F from New England Biolabs), and the extent of digestion was followed through SDS-PAGE. After deglycosylation the protein was re-purified and crystallization was attempted. All the crystallization attempts using the enzymatically de-glycosylated Evasin-1 produced only microcrystals. We therefore decided to express the Evasin-1 in Tn5 cells in the presence of tunicamycin, a N-glycosylation inhibitor [44].

Crystals of non-glycosylated Evasin-1 were obtained by vapour diffusion, using hanging drops, in the presence of 3% PEG 4K, 0.2 M (NH_4_)_2_SO_4_ and 10% methylpentanodiol (MPD). These crystals grow in one week at room temperature up to a maximum size of 0.3×0.3×0.3 mm^3^. The crystals belong to the space group P2_1_2_1_2_1_ with unit-cell dimensions of a = 39.60 Å, b = 46.16 Å and c = 99.59 Å. The solvent content is approximately 39%, with two molecules of Evasin-1 per asymmetric unit (Mathews, 1968). Heavy atom derivatives were screened at different concentrations and with different soaking times, and the most successful derivatives were prepared using 5 mM K_2_PtCl_4_ (24 hours) and 5 mM AuKCl_4_ (24 hours). Upon reaction with the heavy atoms, the crystal diffraction quality decreased significantly presenting severe anisomorphism and anisotropy. All datasets were collected at the X06SA-PXI beamline of the Swiss Light Source (SLS) at the Paul Scherrer Institute (PSI) in Villigen (Switzerland).

Data were indexed and processed using DENZO and SCALEPACK from the HKL package[45]. Initial heavy atom positions were determined by Patterson methods using SHELX[46] and further refined with SHARP[47]. The quality of the initial electron density map was significantly improved by solvent flattening using SOLOMON[48] through the interface in autoSHARP [49]. The results of the phasing calculations are summarized in Table S2.

The initial model was traced with ARP-WARP[50], with 77 out of 200 residues being assigned to the electron density. For graphical interpretation of electron density, we used the software packages O[51], COOT[52] and MAPMAN BONES[53] for the initial electron-density skeletonisation. The initial model was improved by visual inspection and model building with COOT [52]. The model was refined to 1.70 Å resolution using CNX[26], with a final R-value of 22.6% and free-Rvalue of 26.9 (5% test set) using the parameter set of Engh and Huber [54]. The data processing of the high resolution data set and refinement statistics are summarised in Table S3. We have found two monomers (A/B) in the asymmetric unit corresponding to a solvent content of 39.0% (VM  = 2.02 Å^3^ Da^−1^) [25]. The final refined atomic model comprises residues 5–91 of monomer A and residues 10–89 for monomer B, with the missing residues not observed in the electron density (comprising the N-terminal and the C-terminal 6-his tag).

### Crystallization and Structure Determination of the Glycosylated Evasin-1

The first crystals of glycosylated Evasin-1 produced in Tn5 cells were obtained in sitting drops vapour diffusion screenings using the 96 well Crystal Screen HT from Hampton Research. Conditions containing 30% polyethylene glycol (PEG) 4K or PEG 8K and 0.2 M (NH_4_)_2_SO_4_ produced microcrystals. After optimisation hexagonal rod shaped crystals grow up to maximum dimensions of 0.3–0.8 mm in 20 days in 18% PEG 4K and 0.4 M AS, or 10 days in 17% PEG 4K, 0.3 M (NH_4_)_2_SO_4_ and 3% dioxane. These crystals belong to the space group P3_1,2_21 with unit cell a = b = 116.69 Å, c = 58.82 Å, and diffracted up to 3.75 Å using synchrotron radiation. Using a different protein preparation expressed in Sf9 cells, it was possible to obtain cubic pyramid shaped crystals in 23% PEG 4K and 0.3 M (NH_4_)_2_SO_4_. These crystals grow up to 0.2×0.2×0.2 mm^3^ and a complete data set was obtained up to 2.70 Å using synchrotron radiation. These crystals belong to the space group P2_1_2_1_2_1_, with unit cell a = 68.70 Å, b = 70.49 Å, c = 103.82 Å. The crystal structure of the glycosylated version of Evasin-1 was solved later by molecular replacement in AMORE[55] using the non-glycosylated Evasin-1 as a search model. We have found three monomers in the asymmetric unit corresponding to a solvent content of 67% (Mathews, 1968). The model was built using COOT[52] and refined against the 2.70 Å resolution data with CNX[26], with a final R-value of 28.5% and with free-R value of 33.9 (5% test set). The data processing and refinement statistics are summarised in Table S4.

### Crystallization and Structure Determination of the Complex between Evasin-1 and CCL3

The human chemokine CCL3 variant ΔAla^1^-CCL3 (A10T) lacking the first alanine residue and presenting the mutation A10T[56] was produced in *E. coli* BL21 (DE3), and it was expressed and purified as described[57].

The complex between the non-glycosylated Evasin-1 and CCL3 was prepared by incubating Evasin-1 overnight at 4°C with an excess of CCL3, in a molar ratio of 1∶2.5, which was then captured on an (Ni-NTA) affinity column. Complex formation was followed by gel filtration analysis (Superose 12) of the peak fractions and SDS-PAGE, and pooled according to homogeneity of the sample. The fraction containing the complex was then concentrated to 10 mg/ml in 25 mM Tris-HCl pH 8.0, 100 mM NaCl. Crystals of the complex were obtained at room temperature by vapour diffusion, using sitting drops, in the presence of 24% PEG 3350, 0.2 M ammonium sulphate and 0.1 M HEPES pH 8.1. The crystals appear in one month and continue to grow for another month up to a maximum size of 0.2×0.2×0.2 mm^3^. The crystals belong to the space group P4_3_32 with unit-cell dimensions of a = b = c = 104.384 Å. The solvent content is approximately 50.6%, with one 1∶1 heterodimeric complex of Evasin-1 /CCL3 per asymmetric unit[25].

The Evasin-1 position was located by molecular replacement with AMORE[55], using the previously determined Evasin-1 as a search model. The initial molecular replacement phases produced electron density maps of very good quality where the missing CCL3 monomer could be identified by visual inspection, confirming that the correct molecular replacement solution was found. The complex Evasin-1 and CCL3 model was traced using ARP-WARP[50], with 149 out of 169 residues being docked initially to the electron density. After visual inspection with COOT[52] and refinement with CNX[26], the final model has an R-value of 24.2 and a free R-value of 29.9 (5% test set) for all 30–1.90 Å data. The data processing and refinement statistics are summarised in the Table 1. The refined atomic model of the Evasin-1 and CCL3 complex comprises the residues 1–100 of Evasin-1 and 2–67 of CCL3 with the last 2 residues from the C-terminal of CCL3 not being observed.

**Table 1:** Summary of data collection and refinement statistics for the complex.

Data collection	Complex
Space group	P4_3_32
Cell parameters	104.38
Wavelength (Å)	0.976
Resolution (Å)	30.00–1.9
Total observations	649696
Unique reflections	15897
I/σ	44.7 (15.3)
Rsym (%)	8.4 (37.3)
Completeness (%)	99.8 (100.0)
Redundancy	40.9
Refinement statistics	
R_cryst_	24.2
R_free_	29.9
Number of molecules in asymmetric unit	1 heterodimer (A/B)
Number of protein atoms (A/B)	785/524
Number of solvent atoms/Ni atoms	223/3
Rmsd Bond length (Å)	0.004
Rmsd Bond angles (degrees)	0.88
Average B factors	
Protein atoms (A/B) (Å^2^)	31.7/33.3
Solvent molecules/Ni atoms (Å^2^)	51.2/33.5
Ramachandran plot	
Most favored/additional (%)	89.4/8.5
Generous/disallowed (%)	1.4/0.7

### Cloning, Expression and Purification of Chemokine the Chimera CCL3/CXCL8 and CCL5/CCL3

The chemokine chimeras were produced essentially as described elsewhere[58], subcloned into a pET30a expression vector, the protein expressed in *E. coli* BL21(DE3) and the protein purified following standard chemokine techniques[59].

In the first PCR step, the core of CXCL8 for CCL3/CXCL8 or CCL3 for CCL5/CCL3 were amplified to obtain the sequence of the chemokine lacking its N terminus sequence up to the CXC or CC motif, replaced by the five last N terminal amino acids CCL3 or CCL5. The second PCR step generated the full sequence of the N terminus of CCL3 or CCL5. After solubilisation of the inclusion bodies in 6 M guanidine, both CCL3/CXCL8 or CCL5/CCL3 protein solutions were directly renatured by dropwise dilution at 4°C into 0.1 M Tris-HCl pH 8.0 containing 0.1 mM reduced glutathione and 0.01 mM oxidized glutathione, to obtain a final protein concentration of 50 µg/ml. In the case of the CCL5/CCL3, the initiating methionine was retained when the protein is expressed in *E.coli* and was subsequently removed by methionine aminopetidase (MAP) (PeproTech) digestion. CCL5/CCL3 was suspended at 1 mg/ml in 35 mM Tris/HCl pH 8, the MAP was then added at a ratio enzyme: substrate of 1∶1′250 (w:w) and the digestion carried out for 24 h at 37°C. The digested protein was then purified on an analytic RP-HPLC as described previously, quantified by UV at 280 nM, aliquoted and lyophilised.

### Surface Plasmon Resonance Analyses

Real-time biomolecular interaction analyses were performed using a BIAcore 3000 surface plasmon resonance (SPR) system. Chemokine binding analyses were performed on immobilized Evasin-1. Evasin-1 was suspended at 50 µg/ml in 10 mM sodium acetate buffer pH 4.5 and directly immobilized on a CM4 chip (Biacore) by a standard amine coupling chemistry with the Biacore Amine coupling kit (Biacore) according to manufacturer's instructions, to reach a level of 300–400 response units (RU) using the Biacore3000 Wizard software. A blank cell was prepared as a control with the chemical coupling without any added protein. Experiments were performed at 25°C and 30 µl/min using HBS-P running buffer (0.01 M HEPES pH 7.4, 0.15 M NaCl and 0.005% surfactant P20) (Biacore). Chemokines were suspended at 0.1 µg/ml in running buffer and for binding experiments and all protein solutions were filtered through a 0.22 µm filter. The injection time was 3 min followed by a dissociation time of 2.5 min after injection. The chip was regenerated using 50 mM Glycine buffer, pH 2 for 30 s. For each experiment, chemokines were injected in triplicate in random order.

For Evasin-1 WT and F14A W89A binding on immobilized CCL3, the same procedures were applied with the following changes: CCL3 was suspended at 25 µg/ml in 10 mM sodium acetate buffer pH 4 and immobilized on the chip and Evasin-1 WT and F14A W89A were suspended at 1 µg/ml in running buffer for binding experiments.

For the kinetic experiments, 6 dilutions of chemokines or Evasin-1 WT and F14A W89A were prepared in running buffer, filtered through a 0.22 µm filter, and injected over the experimental and blank flow cells. The injection time was 3 min followed by a dissociation time of 15 min and the chip was regenerated using 50 mM Glycine pH 2 buffer for 30 s. Again, each chemokine or Evasin-1 WT and F14A W89A dilution was injected in triplicate in a random order.

For the analysis, the sensograms from the blank cell, in addition to the sensograms obtained with the running buffer alone were subtracted from the binding to remove the system noise. For the kinetics, the association (k_a_) and the dissociation (k_d_) values were determined simultaneously by globally fitting sensograms for an entire range of chemokine concentrations according to the langmuïr-fitting model. The apparent equilibrium dissociation constants (K_d_) were determined from the mean kinetics values with the equation: K_d_ = k_d_/k_a_.

## Supporting Information

Table S1 List of the residues at the interface of the complex and their main interactions. (A) residues from Evasin-1, and (B) residues from CCL3.
(0.01 MB DOC)

Table S2 Data collection and MIRAS Phasing statistics (SHARP)
(0.01 MB DOC)

Table S3 Summary of data collection and refinement statistics for the native dataset 2 of Evasin-1
(0.01 MB DOC)

Table S4 Summary of data collection and refinement statistics for the glycosylated Evasin-1
(0.01 MB DOC)
