# Supplementary material for: Structural Basis of Chemokine Sequestration by a Tick Chemokine Binding Protein: The Crystal Structure of the Complex between Evasin-1 and CCL3
Source: PLoS One. 2009 Dec 30;4(12):e8514. doi: 10.1371/journal.pone.0008514 (PMC2796168; doi:10.1371/journal.pone.0008514)
Supplement: Table S1 — List of the residues at the interface of the complex and their main interactions. (A) residues from Evasin-1, and (B) residues from CCL3. (0.01 MB DOC) [file pone.0008514.s001.doc]

**Table S1. List of the residues at the interface of the complex and their main interactions.** (A) residues from Evasin-1, and (B) residues from CCL3.

| Residue | Polar (Hbonds) | Hydrophobic  (ligplot/hbplus) | Residue | Polar (Hbonds) | Hydrophobic  (ligplot/hbplus) |
| --- | --- | --- | --- | --- | --- |
| **Evasin-1 (A)** |  |  | CCL3 (B) |  |  |
| Asp2A | Ser17B |  | Leu3B |  | Arg90A |
| Asp3A | Ser17B |  | Ala5B | Trp89A, Arg90A | Arg90A, Lys93A |
| Glu4A | Ser17B | Ser17B | Asp6B | Arg86A, Asn88A, Arg90A | Asn88A |
| Asp5A |  | Arg18B | Thr7B | Asn88A, Trp89A | Asn88A |
| Tyr6A | Arg18B | Arg18B | Pro8B |  | Leu54A, Arg55A |
| Gly7A | Arg18B |  | Thr9B | Val16A, Arg87A | Val16A, Glu18A, Asn88A, Trp89A |
| Leu9A | Thr16B | Ile20B | Thr10B |  | Pro13A, Phe14A, Leu15A |
| Gly10A | Arg48B |  | Cys11B | Phe14A | Val16A, Pro24A |
| Gly11A | Cys51B |  | Phe13B |  | Phe14A, Ala40A |
| Cys12A |  |  | Ser14B | Glu38A |  |
| Pro13A |  | Thr10B, Ile41B, Gln49B, Cys51B | Thr16B | Leu9A |  |
| Phe14A | Cys11B | Thr10B, Phe13B | Ser17B | Asp2A, Asp3A, Glu4A | Glu4A |
| Leu15A |  | Thr10B | Arg18B | Tyr6A, Gly7A | Asp5A, Tyr6A |
| Val16A | Thr9B | Thr9B, Cys11B | Ile20B |  | Leu9A |
| Glu18A |  | Thr9B | Phe24B |  |  |
| Thr21A | Gln34B |  | Phe29B |  | Trp89A |
| Gly22A |  | Gln34B | Glu30B |  | Trp89A |
| Tyr23A |  | Gln34B | Thr31B |  |  |
| Pro24A |  | Cys11B, Gln34B, Cys35B | Ser32B |  |  |
| Ile26A |  |  | Gln34B | Thr21A | Gly22A, Tyr23A, Pro24A, |
| Cys33A |  | Arg48B | Cys35B |  | Pro24A |
| Asn34A | Arg48B | Arg48B | Ile41B |  | Pro13A |
| Glu38A | Ser14B |  | Thr44B |  |  |
| Ala40A |  | Phe13B | Arg48B | Gly10A, Asn34A | Cys33A, Asn34A |
| Pro41A |  |  | Gln49B | Trp89A | Pro13A |
| Thr44A |  |  | Val50B |  |  |
| Leu54A |  | Pro8B | Cys51B | Gly11A | Pro13A |
| Arg55A |  | Pro8B |  |  |  |
| Arg86A | Asp6B |  |  |  |  |
| Arg87A | Thr9B |  |  |  |  |
| Asn88A | Asp6B, Thr7B | Asp6B, Thr7B, Thr9B |  |  |  |
| Trp89A | Ala5B, Thr7B, Gln49B | Thr9B, Phe29B, Glu30B |  |  |  |
| Arg90A | Ala5B, Asp6B | Leu3B, Ala5B |  |  |  |
| Lys92A |  |  |  |  |  |
| Lys93A |  | Ala5B |  |  |  |

H-hydrophobic or non-bonded interactions (ligplot/hbplus)

P – Polar or hydrogen bonds (contact/ligplot/hbplus)

Other buried residues – CNX buried interface residues
