# Supplementary material for: Structural Basis of Chemokine Sequestration by a Tick Chemokine Binding Protein: The Crystal Structure of the Complex between Evasin-1 and CCL3
Source: PLoS One. 2009 Dec 30;4(12):e8514. doi: 10.1371/journal.pone.0008514 (PMC2796168; doi:10.1371/journal.pone.0008514)
Supplement: Table S2 — Data collection and MIRAS Phasing statistics (SHARP) (0.01 MB DOC) [file pone.0008514.s002.doc]

**Table S2. Data collection and MIRAS Phasing statistics (SHARP).**

| Data set | Native 1 | K_2_PtCl_4_ | KAuCl_4_ |
| --- | --- | --- | --- |
| Space group |  | P2_1_2_1_2_1_ |  |
| cell parameters | 39.46, 45.80, 99.57 | 39.75, 48.14, 98.07 | 38.86, 48.15, 98.17 |
| Wavelength (Å) | 0.900 | 0.900 | 0.900 |
| Resolution (Å) | 50.00-1.80 | 35.00-2.90 | 35.00-2.70 |
| Total observations | 199763 | 98322 | 362824 |
| Unique reflections | 17247 | 7975 | 9788 |
| I/σ | 15.2 (6.3) | 18.1 (6.3) | 14.3 (5.8) |
| Rsym (%) | 5.3 (27.7) | 5.3 (26.8) | 6.6 (39.0) |
| Completeness (%) | 99.2 (95.7) | 97.9 (100) | 99.0 (100) |
| Redundancy | 5.1 | 3.8 | 4.7 |
| Heavy atom sites |  | 2 Pt | 2 Au |
| R_cullis_ centric/acentric |  | 0.70/0.64 | 0.65/0.64 |
| Anomalous R_cullis_ |  | 0.95 | 0.87 |
| Phasing power centric/acentric |  | 0.46/0.62 | 0.54/0.59 |
| Anomalous Phasing Power |  | 0.71 | 0.71 |
| FOM SHARP | 0.36 |  |  |
| FOM after solvent flattening  and phase extension to 1.80 Å | 0.89 |  |  |
