# Supplementary material for: Structural Basis of Chemokine Sequestration by a Tick Chemokine Binding Protein: The Crystal Structure of the Complex between Evasin-1 and CCL3
Source: PLoS One. 2009 Dec 30;4(12):e8514. doi: 10.1371/journal.pone.0008514 (PMC2796168; doi:10.1371/journal.pone.0008514)
Supplement: Table S3 — Summary of data collection and refinement statistics for the native dataset 2 of Evasin-1 (0.01 MB DOC) [file pone.0008514.s003.doc]

**Table S3. Summary of data collection and refinement statistics for the native dataset 2 of Evasin-1.**

| Data collection | Native 2 |
| --- | --- |
| Space group | P2_1_2_1_2_1_ |
| Cell parameters | 39.60, 46.16, 99.59 |
| Wavelength (Å) | 1.000 |
| Resolution (Å) | 26.0-1.70 |
| Total observations | 164632 |
| Unique reflections | 20431 |
| I/σ | 27.3 (5.1) |
| Rsym (%) | 6.8 (33.3) |
| Completeness (%) | 98.1 (87.4) |
| Redundancy | 8.1 (5.4) |
| Refinement statistics |  |
| R_cryst_ | 22.6 |
| R_free_ | 26.9 |
| Number of molecules in asymmetric unit | 1 homodimer (A/B) |
| Number of protein atoms (A/B) | 655 / 600 |
| Number of solvent atoms | 260 |
| Rmsd Bond length (Å) | 0.006 |
| Rmsd Bond angles (degrees) | 1.10 |
| Average B factors |  |
| Protein atoms (A/B) (Å^2^) | 34.8/36.9 |
| Solvent molecules (Å^2^) | 56.5 |
| Ramachandran plot |  |
| Most favored/additional (%) | 86.5/12.0 |
| Generous/disallowed (%) | 0.8/0.8 |
