# Supplementary material for: Structural Basis of Chemokine Sequestration by a Tick Chemokine Binding Protein: The Crystal Structure of the Complex between Evasin-1 and CCL3
Source: PLoS One. 2009 Dec 30;4(12):e8514. doi: 10.1371/journal.pone.0008514 (PMC2796168; doi:10.1371/journal.pone.0008514)
Supplement: Table S4 — Summary of data collection and refinement statistics for the glycosylated Evasin-1 (0.01 MB DOC) [file pone.0008514.s004.doc]

**Table S4. Summary of data collection and refinement statistics for the glycosylated Evasin-1.**

| Data collection | Complex |
| --- | --- |
| Space group | P2_1_2_1_2_1_ |
| Cell parameters | 68.70, 70.49, 103.82 |
| Wavelength (Å) | 0.979 |
| Resolution (Å) | 58.00-2.70 |
| Total observations | 699350 |
| Unique reflections | 26472 |
| I/σ | 15.2 (3.60) |
| Rsym (%) | 8.0 (40.0) |
| Completeness (%) | 98.9 (98.0) |
| Redundancy | 4.7 |
| Refinement statistics |  |
| R_cryst_ | 28.5 |
| R_free_ | 33.9 |
| Number of molecules in asymmetric unit | 3 |
| Number of protein atoms (A/B/C) | 680/605/622 |
| Number of disaccharide atoms (A/B/C) | 30/15/45 |
| Number of solvent atoms | 59 |
| Rmsd Bond length (Å) | 0.006 |
| Rmsd Bond angles (degrees) | 1.00 |
| Average B factors |  |
| Protein atoms (A/B/C) (Å^2^) | 52.1/58.0/55.0 |
| Disaccharide atoms (A/B/C) (Å^2^) | 93.4/97.2/115.6 |
| Solvent molecules (Å^2^) | 61.5 |
| Ramachandran plot |  |
| Most favored/additional (%) | 82.5/15.0 |
| Generous/disallowed (%) | 1.5/1.0 |
